# AbaI/AbaR Quorum Sensing in *Acinetobacter baumannii*: Virulence Regulation, Epithelial Injury, Innate Immune Activation and Anti-Virulence Strategies

**DOI:** 10.3390/ijms27209212

**Published:** 2026-10-16

**Authors:** Tomasz Busłowicz, Karolina Pawłuszkiewicz, Zofia Biel, Emilia Maria Nowak, Matylda Korgiel, Bachar Cheaib, Bindu Subhadra, Emil Paluch

**Affiliations:** 1 Student Research Group “Medical Image Analysis and Research in Pathology” (SKN MIARP), Faculty of Medicine, Wroclaw Medical University, Wybrzeże L. Pasteura 1, 50-367 Wroclaw, Poland; tomasz.buslowicz@student.umw.edu.pl (T.B.); karolina.pawluszkiewicz@student.umw.edu.pl (K.P.); zofia.biel@student.umw.edu.pl (Z.B.); matylda.korgiel@student.umw.edu.pl (M.K.); 2 Department of Microbiology, Faculty of Medicine, Wroclaw Medical University, Tytusa Chalubinskiego 4, 50-376 Wroclaw, Poland; emilia.nowak@student.umw.edu.pl; 3 Translational Lung Research Center Heidelberg (TLRC-H), German Center for Lung Research (DZL), Heidelberg, Germany, Medical Faculty Heidelberg, Department of Infectious Diseases, Medical Microbiology and Hygiene, Heidelberg University, Im Neuenheimer Feld 672, 69120 Heidelberg, Germany; bachar.cheaib@med.uni-heidelberg.de; 4 Department of Biological and Earth Sciences, Arkansas Tech University, Russellville, AR 72801, USA; bindu.subhadra@gmail.com

**Keywords:** *Acinetobacter baumannii*, quorum sensing, quorum quenching, biofilm, virulence

## Abstract

*Acinetobacter baumannii* (*A. baumannii*) is an important cause of healthcare-associated infections due to its remarkable capacity for environmental persistence and multidrug resistance. The quorum-sensing (QS) network, particularly the AbaI/AbaR system, contributes to the regulation of several traits involved in colonization and infection. Current evidence indicates that AbaI/AbaR-mediated signalling coordinates biofilm formation, surface-associated motility, antimicrobial tolerance via modulation of efflux pump activity, oxidative stress responses and membrane integrity, as well as biofilm-associated antibiotic resistance and stress adaptation. QS also influences interactions with host cells, including epithelial damage and modulation of innate immune responses, although its role in adaptive immunity remains largely unresolved. This review summarises current knowledge of the molecular mechanisms underlying AbaI/AbaR-mediated signalling, its role in regulating *A. baumannii* infection-relevant phenotypes and host–pathogen interactions. It also discusses the therapeutic potential of quorum-quenching (QQ) strategies for attenuating *A. baumannii* virulence, as well as their current limitations in infection control.

## 1. Introduction

*Acinetobacter baumannii (A. baumannii)* is a Gram-negative coccobacillus that has emerged as one of the most problematic opportunistic pathogens in modern healthcare [1,2]. It is responsible for a wide range of healthcare-associated infections (HAIs), including hospital-acquired pneumonia (HAP), ventilator-associated bacterial pneumonia (VABP), bloodstream infections (BSIs), urinary-tract infections (UTIs), meningitis, and skin and soft-tissue infections, predominantly in critically ill hospitalized and immunocompromised patients [3,4,5]. Its capacity for long-term persistence is largely attributable to its robust biofilm-forming ability on medical devices and hospital surfaces, which enhances resistance to antibiotics, disinfectants, and host immune defences [6,7].

A major factor underlying the clinical success of *A. baumannii* is its remarkable capacity to accumulate antimicrobial resistance determinants. Resistance arises through a combination of intrinsic and acquired mechanisms, including the production of OXA-type carbapenemases and metallo-β-lactamases, reduced outer-membrane permeability resulting from porin alterations, multidrug efflux pumps, target-site modification, and the acquisition of resistance genes via plasmids, integrons, and transposons [8,9].

*A. baumannii* is one of the ESKAPE pathogens, a group of clinically important bacteria comprising *Enterococcus faecium*, *Staphylococcus aureus*, *Klebsiella pneumoniae*, *Pseudomonas aeruginosa*, and *Enterobacter* spp., known for their multidrug resistance (MDR) and responsible for a substantial proportion of HAIs worldwide [10]. The World Health Organization (WHO) classification identifies carbapenem-resistant *A. baumannii* (CRAB) as a critical-priority pathogen, underscoring the urgent need for new therapeutic options, antimicrobial stewardship, and strengthened infection-control strategies [11,12]. In 2019, the Centres for Disease Control and Prevention (CDC) designated CRAB as an urgent threat, highlighting its limited treatment options, high mortality, and potential to cause widespread outbreaks in hospitals and long-term care facilities [12,13].

The treatment of resistant *A. baumannii* infections remains highly challenging, as current antibiotic options are limited by nephrotoxicity, suboptimal pharmacokinetic characteristics, inadequate penetration into pulmonary tissues and inconsistent clinical outcomes. An agreed first-line standard of care has therefore not been established [14,15,16]. Consequently, adjunctive and alternative strategies, including phage therapy, nanoparticles, antimicrobial peptides and anti-virulence interventions that target bacterial pathogenicity, are being investigated to overcome the limitations of conventional antibiotics [17,18,19,20,21].

Among the anti-virulence approaches currently under investigation, targeting quorum sensing (QS) has attracted considerable attention because it attenuates bacterial pathogenicity without directly affecting bacterial viability. QS is a cell-density-dependent communication system in which accumulating autoinducers (AIs) activate target gene expression after reaching a threshold concentration [22,23]. This density dependence enables bacteria to postpone metabolically expensive collective behaviours until they become energetically favourable, facilitating the transition from individual activity to coordinated community behaviour. Current anti-QS strategies include quorum quenching (QQ), which degrades or inactivates signalling molecules, and quorum sensing inhibitors (QSIs), which disrupt signal synthesis, receptor binding, or downstream signal transduction [24].

In Gram-negative bacteria, QS is commonly mediated by N-acyl-homoserine lactone (AHL) signalling molecules and LuxI/LuxR-type regulatory proteins [24,25]. In *A. baumannii*, this canonical signalling architecture is represented by the AbaI/AbaR circuit, in which AbaI functions as the AHL synthase and AbaR as the cognate transcriptional regulator. The predominant AI identified in *A. baumannii* isolates is N-(3-hydroxydodecanoyl)-L-homoserine lactone (OHC12-HSL), although the type and quantity of AHLs produced may vary depending on the strains [25,26,27]. Activation of the AbaI/AbaR circuit regulates the expression of genes involved in biofilm formation, surface-associated motility, bacterial persistence, and host colonization, thereby promoting adaptation to the host environment and enhancing pathogenicity [7,25,26,27]. QS influences the expression of factors involved in bacterial adhesion (OmpA, Chaperone-usher system), epithelial cell invasion (*Plc1, Plc2*), and survival in host tissues (biofilm production, *bauA*). Clinical and experimental studies have linked an intact AbaI/AbaR system to increased epithelial cell invasion and enhanced virulence, suggesting that the QS system contributes to epithelial damage during infection [27]. Furthermore, AbaI/AbaR-associated biofilm formation and virulence-related phenotypes may influence host responses during infection, although the direct immunological consequences of this regulation remain incompletely defined [7,25,26,27,28].

This review summarizes the current understanding of the AbaI/AbaR QS system in *A. baumannii*, its contribution to biofilm formation, pathogenicity, epithelial injury, innate immune activation and the emerging potential of QQ enzymatic strategies as adjunct approaches for controlling multidrug-resistant infections.

## 2. Molecular Basis of Abai/Abar QS System

### 2.1. AbaI-Dependent AHL Synthesis

#### 2.1.1. AbaI as the LuxI-Type Autoinducer Synthase

The AbaI/AbaR signalling axis characteristic of *A. baumannii* belongs to LuxI/LuxR-type QS systems, which occur in many Gram-negative bacteria [29,30,31]. The nomenclature of this signalling scheme derives from the model LuxI/LuxR system, first discovered in *Vibrio fischeri*, in which this system controls the expression of the luminescence operon, while the LuxI and LuxR proteins function as an acyl-HSL synthase and a transcriptional activator, respectively [30]. Analogously, AbaI represents the *A. baumannii* LuxI-type autoinducer synthase, whereas AbaR denotes a LuxR-type transcriptional regulator [32,33]. The indirect identification of the AbaI protein was made possible through a clinical *Acinetobacter* spp. isolate producing diffusible molecules capable of activating the biosensor strain *Agrobacterium tumefaciens*, which responds to the presence of AHL [32,33,34]. In the next step, it was demonstrated that a single open reading frame designated *abaI* caused AHL production in recombinant *Escherichia coli* cells [32,34]. Later studies on *A. baumannii* American Type Culture Collection (ATCC) 17978 showed an analogous relationship, as mutation (deletion) of *abaI* abolished AHL production under the examined conditions [34].

Considering AbaI at the level of biochemical features, this protein should be classified as a LuxI-type AHL synthase [32,33]. This type of enzyme is responsible for catalysing the formation of AHL from two substrates belonging to the category of metabolites of cellular processes: S-adenosyl-L-methionine, which provides the homoserine lactone ring, and acylated acyl carrier protein (acyl-ACP), which donates the acyl side chain. Therefore, AHL production is not a simple reaction of producing signalling molecules, but rather a process linked to the basic metabolism of the organism and fatty acid biosynthesis [35]. Additionally, AHL should be considered as a heterogeneous group of signalling molecules, in which differences result from diverse acyl groups of acyl-ACP [35,36]. The structural diversity of AHL molecules is biologically relevant, because variation in the acyl side chain may affect receptor binding, transcriptional activation, and consequently, the phenotype regulated by this QS system [30,35,37]. The dominance of specific AHL variants is influenced by AbaI itself, whose active site may determine the predominance of a product of a particular type [35,36]. Considering *A. baumannii* and other closely related species of the genus *Acinetobacter* spp., AbaI-dependent signalling is dominated by AHLs with long side chains [33,38]. In the case of the widely studied *A. baumannii* strain ATCC 17978, based on genomic analyses and experimental observations, it is now well established that AbaI is the main classical LuxI-type AHL synthase [33,34]. Although mutation of *abaI* in ATCC 17978 abolished detectable AHL production, the presence of LuxI-independent AHL synthesis pathways in other *A. baumannii* strains cannot be completely excluded [34,39].

It should be noted that some of the early insights into AbaI were obtained using the historically important strain referred to as *A. baumannii* M2, which, because of genome analyses, was later reclassified as *A. nosocomialis* M2 [33,40]. Although the findings from the above-mentioned studies do not undermine the key assumptions regarding the mechanism of AbaI action, they should be now presented as evidence derived from species within the *A. calcoaceticus*-*A. baumannii* complex (ACB), a group of closely related species that also includes *A. baumannii* [33,40]. Information directly referring to *A. baumannii*, derived from studies on strains such as ATCC 17978, ATCC 19606, AB5075, and clinical isolates, supported the earlier assumptions resulting from studies on strain M2 [31,33,34,38,41,42].

In terms of the functional aspect of this QS system, the AbaI synthase is responsible for initiating the signal by producing AHLs [32,41]. As a result of AbaI activity, AHLs can be detected in the culture medium, while their distribution between the cell, membrane-associated compartment, and extracellular environment most likely depends on their physicochemical properties [43,44]. AHLs with shorter acyl chains are considered capable of passive diffusion across bacterial membranes, whereas long-chain AHLs interact more strongly with the lipid phase and therefore may potentially exhibit slower distribution between the cell and the environment. However, this distribution model should be regarded as an inference based on general AHL properties rather than as a process directly demonstrated for the AbaI/AbaR system in *A. baumannii* [43,44]. When the signal of this QS system reaches an effective threshold, dependent on the concentration and properties of the AHL, population density, and culture conditions, the signalling molecule binds to AbaR, which acts as a cytoplasmic AHL-responsive transcriptional regulator, although the structure of this binding site has not yet been resolved [33,37,39,41,43]. This binding prompts the AbaR-AHL complex to interact with specific promoter regions, typically described as lux box-like motifs, directly causing broad transcriptional changes, including positive feedback involving *abaI* expression [32,41,42]. However, the precise characteristics of this AbaR binding site, including its exact sequence length and a validated consensus motif, remain poorly defined. Furthermore, while the *abaR* gene itself is highly conserved across *A. baumannii* species, the sequence-level conservation of its specific DNA-binding operator has not yet been systematically characterized [32].

#### 2.1.2. Chemical Nature and Diversity of AHL Signals

At the molecular level, AHLs consist of a conserved homoserine lactone ring connected by an amide bond to an acyl chain, which varies with respect to length, degree of saturation, and degree of oxidation at the C3 position, including unsubstituted, 3-hydroxy, and 3-oxo forms [37,45]. Elongation of the acyl chain increases the hydrophobicity of the molecule, its interactions with membranes and its stability against lactonolysis [44,46]. These are biochemically important differences that affect signal properties such as hydrophobicity, diffusion, partitioning into the membrane compartment, stability, and selectivity of receptor binding [37,44,45,46]. Accordingly, in LuxI/LuxR-type systems, the acyl chain co-determines the specificity and kinetics of signalling, because the corresponding LuxR-type regulators usually contain a binding domain matched to an acyl chain of a defined length and corresponding substitution profile [37,45].

This is particularly important in the case of *Acinetobacter* spp., because many examined strains belonging to the ACB complex predominantly produce long-chain AHLs, particularly molecules with C10-C14 acyl chains [33,34]. Among the AHL signals identified in *A. baumannii*, OHC12-HSL is the best-characterized to date [33,34]. In studies on *A. baumannii* ATCC 17978 using high-performance liquid chromatography coupled with mass spectrometry, OHC12-HSL was identified as the main AHL type detected in the supernatants of static cultures [33,34].

When analysing the diversity of the AbaI-dependent signal specifically in *A. baumannii*, the available evidence provided by Mayer et al. also supports the presence of additional AHLs [33]. The full chemical names and abbreviations of AHL Signals Discussed in the AbaI/AbaR QS System of A. baumannii are listed in Table 1.

In addition to the dominance of OHC12-HSL in ATCC 17978, smaller amounts of N-(3-hydroxydecanoyl)-L-homoserine lactone (OHC10-HSL), N-(3-oxododecanoyl)-L-homoserine lactone (OC12-HSL), and N-(3-hydroxytetradecanoyl)-L-homoserine lactone (OHC14-HSL) were also detected. Under some culture conditions, small amounts of N-hexanoyl-L-homoserine lactone (C6-HSL), N-(3-oxohexanoyl)-L-homoserine lactone (OC6-HSL), and N-octanoyl-L-homoserine lactone (C8-HSL) were also recorded. Information from studies on clinical isolates also indicates heterogeneity through the detection of OHC10-HSL, OC12-HSL, N-(3-oxotetradecanoyl)-L-homoserine lactone (OC14-HSL), and OHC14-HSL. It should be noted that in all clinical isolates examined in that study, the *abaI* and *abaR* genes were amplified by polymerase chain reaction (PCR), and their sequences showed high identity to the corresponding genes in strain ATCC 17978. Therefore, the heterogeneity of the AHL profile is probably not solely dependent on the presence of the canonical AbaI/AbaR genes but may result from differences in the regulation of their expression, the activity of QQ enzymes, nutrient availability, or culture conditions [33].

#### 2.1.3. Factors Influencing AHL Accumulation

The classical QS system model describes AHL accumulation as a process dependent on increasing bacterial density until a functional level is reached, which is necessary to activate receptor-dependent signalling pathways [29,37,39]. In the case of *A. baumannii*, a strictly defined numerical threshold for AbaR activation by AHLs has not been established. In the literature devoted to AbaI-dependent AHL production, experimentally observed patterns of accumulation of these signalling molecules are primarily presented [33,34,38]. The best characterization of these trends has been described based on information derived from strain ATCC 17978 [33,34]. Under static culture conditions, OHC12-HSL accumulation in ATCC 17978 was growth-phase dependent, peaking near the transition to stationary phase and declining sharply thereafter. Attention should also be paid to the different results obtained in shaken cultures, where *A. baumannii* populations showed low *abaI* expression and no detectable AHLs in the tested media. These observations support the conclusion that static conditions possibly reflecting surface-associated growth or increased cell-to-cell contact favour AbaI-dependent QS signalling [33,34,42]. AHL accumulation may also be affected by variability within the species itself [27,33]. Among 80 *A. baumannii* clinical isolates, *abaI* and *abaR* were common, but only 24 of 61 isolates carrying both genes produced biosensor-detectable AHLs. This shows that the presence of *abaI* and *abaR* does not necessarily predict an active AHL-producing phenotype [27].

Additional factors determining AHL signal accumulation are environmental conditions [33,34,47]. Both in the case of strain ATCC 17978 and in studies on clinical isolates, the profile of produced AHLs depended on nutrient availability or on the salt concentration in the medium [33,34]. Blue light and temperature were also identified as environmental elements modulating, in this case, *abaI* expression and AHL production in ATCC 17978 [47,48]. At 23 °C, *abaI* expression was markedly higher in darkness than under blue light, and AHLs were mainly detected in dark-grown biofilm-associated samples. At 37 °C, the direction was reversed. AHL production and *abaI* expression were higher under blue light than in darkness, although the magnitude of the light-dark difference was smaller than at 23 °C [47].

#### 2.1.4. Consequences of *abaI* Disruption

The consequence of deletion or inactivation of *abaI*, which prevents functional AbaI production, is impairment of AbaI-dependent AHL synthesis below the detectable level, which translates into disruption of the AHL-dependent component of AbaI/AbaR system signalling [34,38,42]. In *A. baumannii* ATCC 17978, deletion of *abaI* abolished AHL production and reduced both surface-associated motility and biofilm formation [34]. Sun et al. present that *abaI* deletion was associated with reduced surface-associated motility, altered biofilm formation, decreased serum resistance and virulence [38].

Although AbaI-dependent AHL production is a key element of AbaI/AbaR signalling, the impact of *abaI* disruption extends beyond the simple disappearance of the extracellular signal [41,42,49,50]. RNA sequencing of the *ΔabaI* mutant of ATCC 19606 revealed 380 genes with altered expression. This group included 256 upregulated genes and 124 downregulated genes. Enrichment analysis showed downregulation of pathways associated with arginine biosynthesis, purine metabolism, biofilm formation, and the type VI secretion system (T6SS), whereas pathways related to fatty-acid and amino-acid metabolism were upregulated [41]. This supports a model in which AbaI/AbaR signalling participates directly or indirectly in a broad spectrum of transcriptional processes, rather than only in selected virulence traits [41,42,49,50]. However, it is important to interpret the transcriptomic changes observed after *abaI* deletion as a consequence of disruption of the AbaI/AbaR system and not as evidence that every gene with altered expression is a direct transcriptional target of AbaR [37,41,42]. Xiong et al. noted that RNA-sequencing studies performed in *abaI* deletion backgrounds in ATCC 17978, AB5075 and clinical strains showed different numbers of differentially expressed genes, partly because of different analytical cutoffs and culture conditions [41,42]. Some changes may reflect direct AbaR-dependent regulation, although direct promoter binding has not been demonstrated for most of the identified genes [37,41].

Recent proteomic data further support the view that *abaI* disruption results in extensive remodelling of the bacterial cell at transcriptomic and proteomic level [41,42,49]. In the *ΔabaI* mutant of ATCC 17978, altered expression of polypeptides associated with membrane proteins, membrane transport, membrane biogenesis, and antibiotic resistance was demonstrated. The mutant exhibited reduced levels of outer-membrane-associated proteins, including Survival Protein A (SurA), an Outer Membrane Protein A (OmpA) family protein, an Outer Membrane Protein W (OmpW) family protein, and β-Barrel Assembly Machinery Protein A (BamA), linking *abaI* disruption to changes in cell envelope organization and potentially increased vulnerability to membrane-associated stresses [49,51].

### 2.2. AbaR-Mediated Transcriptional Regulation and Positive-Feedback Loop

AbaR is a 198-amino-acid transcriptional receptor-regulator belonging to the LuxR-type receptor family [25,32,37,52,53]. It constitutes a component of the AbaI/AbaR QS system that links AbaI-dependent production of AHLs with corresponding transcriptional changes in the studied *A. baumannii* strains [25,31,38,41,42,50,52,53]. AbaR is expected to contain an N-terminal domain responsible for AHL binding and a C-terminal helix-turn-helix domain predicted to mediate DNA binding at regulated promoter regions [37,39].

The *abaI* promoter region remains the most frequently discussed candidate site for AbaR-mediated feedback regulation. In the historically studied M2 strain the nucleotide sequence CTGTAAATTCTTACAG, located 67 bp upstream of the predicted *abaI* start codon, was described as a Lux-box-like sequence and proposed as a putative binding site through which AbaR could participate in the autoinductive positive-feedback loop of *abaI* [32,33,40]. In *A. baumannii* AB5075, a putative Lux-box-like sequence has also been indicated in the intergenic region between *abaM* and *abaI*, upstream of the predicted *abaI* start codon, within the broader *abaR*-*abaM*-*abaI* locus architecture [42]. However, these candidate regions should not be regarded as validated AbaR-binding sites. To date, the complete regulatory sequence recognized by AbaR has not been directly mapped, and no experimental structure of the protein has been obtained. Although later studies successfully generated *abaR* mutants and showed that AbaR affects *abaI* expression and biofilm-related phenotypes, the structural basis of AbaR-AHL-DNA recognition and the direct promoter occupancy of AbaR remain unresolved [25,32,42,52,53]. Nevertheless, the presence of the LuxR-type *abaR* gene within the *abaI*-associated locus, together with candidate Lux-box-like sequences upstream of *abaI* and functional data from *abaR* mutants, supports AbaR as the most plausible cognate regulator mediating this feedback [32,42,52,53].

Positive feedback within the AbaI/AbaR system was demonstrated in a study using strain M2, a representative of the closely related ACB complex [25,32,33,40]. Evidence supporting the positive-feedback-loop model directly in *A. baumannii* is based on strain AB5075, in which an *abaI* promoter-lux reporter fusion was used to indirectly demonstrate an increase in *abaI* promoter activity stimulated by exogenous OHC12-HSL. In addition to increased *abaI* expression, the same study also showed that *abaM* expression is positively regulated by QS, whereas AbaM functions as a negative modulator of the QS circuit, since disruption of *abaM* increased AHL production and *abaI*/*abaR* expression [42]. Thus, in *A. baumannii*, AbaR-associated autoinduction of *abaI* is best viewed as part of a broader feedback architecture rather than as a simple linear loop [25,32,42,52,53].

The effects of AbaR-associated gene-expression regulation within the AbaI/AbaR QS system seem to extend beyond this feedback mechanism and modulate numerous characteristics, including metabolism, virulence-associated traits, stress responses, cell envelope organization, and surface-associated growth [38,41,42,49,50,51,52,53]. However, the direct AbaR regulon has not yet been comprehensively defined, and many reported downstream or upstream effects of AbaI/AbaR signalling are inferred from studies of *abaI* mutants, AHL-production status, or global expression analyses rather than from evidence of direct AbaR-promoter binding [41,42,49,50,52,53]. This makes it difficult to distinguish the primary effects of the QS system from its secondary effects [41,42,50,52,53].

### 2.3. AbaM-Dependent Modulation and Strain-Dependent Complexity

The current perception of the AbaI/AbaR QS system in *A. baumannii* should extend beyond a simple two-gene LuxI/LuxR-type system [25,31,37,42]. In *A. baumannii* AB5075 studies identified *abaM* as a third gene located between *abaR* and *abaI*, with *abaR* divergently transcribed relative to the tandemly oriented *abaM* and *abaI* genes, and a putative Lux-box-like sequence located in the intergenic region between *abaM* and *abaI*, upstream of the predicted *abaI* start codon. Comparative genomic analysis indicated that this three-gene locus organization is conserved among *Acinetobacter* spp. [42]. The product of this gene, AbaM, is a small RsaM-type protein (the first representative of this family of AHL-dependent QS systems negative regulators was originally identified in *Pseudomonas fuscovaginae*) that is predicted to function as neither an AHL synthase nor a LuxR-type AHL receptor within the AbaI/AbaR system [42,54,55]. Such organization of the locus is important from the perspective of refining the mechanistic model of the described QS system and the interdependent modulation of the expression of its constituent genes. Experimental findings have suggested that stimulation with OHC12-HSL enhances the expression of both *abaI* and *abaM*, resulting in a more diverse response that extends beyond the simplest model of a positive-feedback loop [42,55].

**Figure 1:**
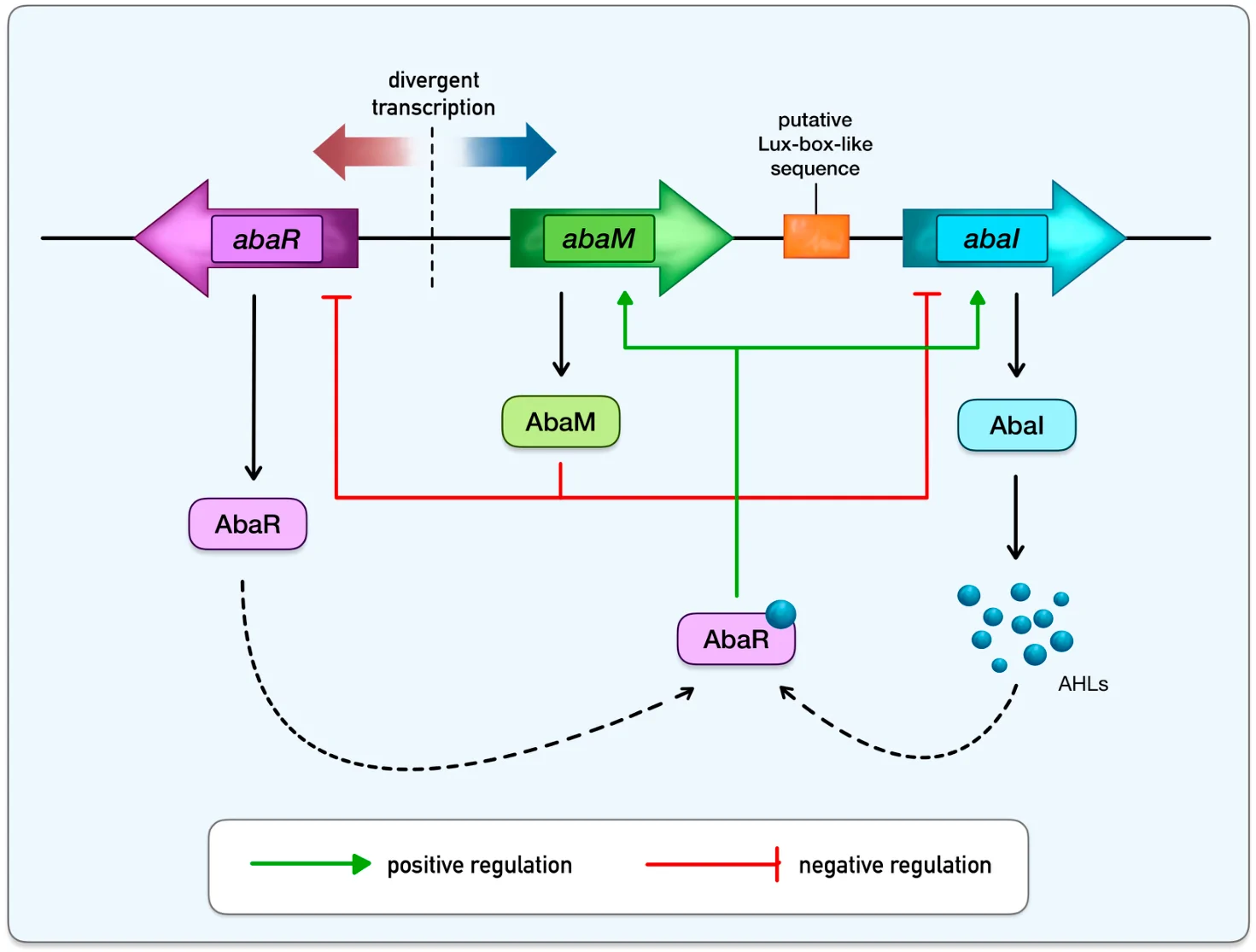
Proposed model of the *abaR*-*abaM*-*abaI* locus architecture and simplified autoregulatory interactions within the AbaI/AbaR/AbaM quorum-sensing (QS) system [31,32,37,38,42,52,53,54,55]. Gene and corresponding protein are represented by the same colour: *abaR*/AbaR (purple), *abaM*/AbaM (green), and *abaI*/AbaI (cyan). Green arrows indicate positive regulation, red lines with terminal bars indicate negative regulation, solid black arrows indicate gene expression/protein production, and dashed black arrows indicate proposed regulatory interactions.

Experimental data, particularly those obtained from strain AB5075, support the RsaM-like nature of AbaM and characterize this protein as a negative regulator of the AbaI/AbaR system [42,55]. Disruption of AbaM was shown to cause a significant increase in AHL production and increased expression of *abaI* and *abaR*, supporting the view that AbaM restricts the activity of the signal-production and signal-reception components of the pathway rather than constituting an additional signal-producing or signal-receiving module [42]. The currently accepted organization of the *abaR*-*abaM*-*abaI* locus and the proposed autoregulatory interactions within this module are summarized in Figure 1 [31,32,37,38,42,52,53,54,55]. Additionally, AbaM is considered to be more than merely a minor regulatory element of the QS pathway. Transcriptomic analysis showed that AbaM modulates approximately 21% of the regulon of the AbaI/AbaR system. However, similarly to the reservations concerning the assessment of the direct and indirect effects of AbaR stimulation, in the case of AbaM it also remains unknown what proportion of the modulation of the transcriptional program results from the direct action of the *abaM* product and what proportion constitutes a secondary effect of the mechanism restricting the activity of the AbaI/AbaR signalling system itself [42,55].

Important information regarding the functional consequences of AbaM activity was provided by phenotypic observations in *abaM* mutants. Loss of AbaM was associated with increased biofilm formation and surface-associated motility, as well as significantly reduced virulence in a *Galleria mellonella* infection model [42]. These paradoxical observations of enhanced QS signalling and surface-associated phenotypes show that these effects should not necessarily be equated with increased virulence in vivo, while the AbaM regulator may potentially act as a factor preventing excessive AbaI/AbaR activity and thereby contribute to maintaining QS signalling at a desirable level, which may favour the preservation of pathogenic fitness [38,42,55].

AbaM-mediated modulation may also explain why the AbaI/AbaR system does not produce identical effects in all *A. baumannii* strains or isolates. AHL accumulation is additionally controlled by AbaM, which negatively regulates the AbaI/AbaR system and may reduce AHL levels below the detection threshold of some assays. The mere presence of *abaI* and *abaR* is not equivalent to detectable AHL production, while detectable AHL production should not be equated with a uniform pathogenic phenotype [27,33,42]. Studies of clinical isolates indicate that some isolates possessing both canonical genes of the discussed QS system do not produce AHLs detectable using a biosensor, whereas other isolates producing detectable AHLs vary in terms of surface-associated motility, invasion capacity, behaviour in virulence models, and biofilm formation [27]. This suggests that the *abaI*/*abaR* genotype, AHL production, and downstream phenotype should be regarded as related but distinct functional levels of the same QS system [27,33,42].

In addition to differences in the expression of *abaI*, *abaR* and *abaM*, strain-dependent variability may also be influenced by variation in the architecture of the promoters of both the canonical genes and those belonging to the QS-system regulon, the structure of AHLs and the rate of their degradation, the metabolic state of the cells, environmental growth conditions, and interactions with other regulatory pathways [27,32,33,37,41,42,44,45,46,47,48,50,52,53,55]. Therefore, AbaI/AbaR/AbaM signalling appears to be part of a broader adaptive signalling network rather than an isolated mechanism determining specific virulence traits [27,31,41,42,49,50,55].

Because evidence for the AbaI/AbaR/AbaM QS system has been derived from different experimental models and strain backgrounds, direct generalization across studies requires caution. The historically important M2 strain, reference *A. baumannii* strains, the AB5075 model and clinical isolate collections each contributed different types of evidence and have distinct interpretative limitations. These key study models and their relevance to the current understanding of AbaI/AbaR/AbaM signalling are summarized in Table 2 [27,32,33,34,40,41,42,49].

## 3. AbaI/AbaR-Regulated Phenotypes Relevant to *A. baumannii* Infection

**Figure 2:**
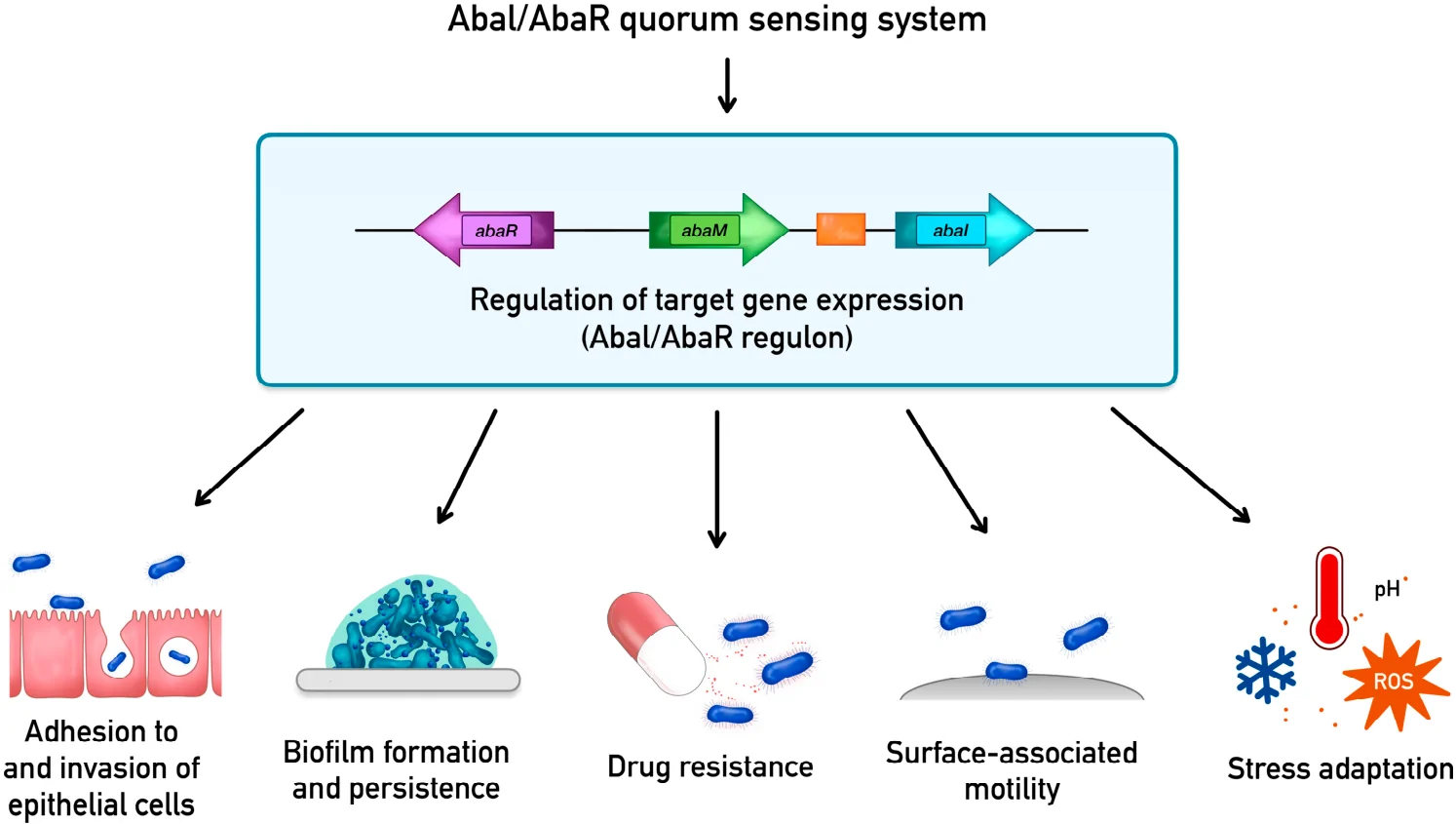
Infection-associated functions associated with the AbaI/AbaR quorum sensing (QS) system in *Acinetobacter baumannii* [31,56,57,58]. Abbreviations: ROS—reactive oxygen species.

Figure 2 presents the role of AbaI/AbaR-regulated phenotypes relevant to *A. baumannii* infection like biofilm formation and persistence, surface-associated motility, adhesion to and invasion of epithelial cells as well as drug resistance and stress adaptation [31,56,57,58].

### 3.1. Role of QS in Biofilm Formation and Persistence of A. baumannii

One of the major determinants enabling *A. baumannii* to persist in healthcare-associated environments and contribute to infectious disease is its capacity to form biofilms, defined as highly organized bacterial communities within a self-produced extracellular polymeric substance (EPS) matrix [59,60,61]. In a study evaluating 100 *A. baumannii* isolates from immunocompromised patients hospitalized in an intensive care unit (ICU), all analysed isolates demonstrated the ability to form biofilm [62]. Furthermore, biofilm formation is estimated to contribute to over 65% of HAIs and nearly 80% of chronic infections [62,63]. The QS system is recognized as an important regulatory mechanism controlling biofilm development and virulence regulation in *A. baumannii* [64].

Biofilm formation in *A. baumannii* is a multifactorial process driven by several genes that support bacterial adhesion, surface colonization and biofilm maturation. Among the most frequently described determinants are *ompA*, which encodes *Acinetobacter* OmpA (AbOmpA), beta-lactamase PER-1 (*blaPER-1*) and biofilm-associated protein (*Bap*) [65]. The role of OmpA has been demonstrated in *A. baumannii* ATCC 19606. Strains lacking *ompA* showed a reduced ability to form biofilm, whereas replenishment of a functional *ompA* gene recovered this phenotype [66]. At the same time, genomic studies of extensively drug-resistant (XDR) *A. baumannii* isolates indicate that biofilm-associated genes may vary substantially between strains [67]. Recent studies suggest that understanding the molecular mechanisms of biofilm formation may help evaluate new therapeutic strategies against *A. baumannii*. Yang et al. showed that α-pinene combined with meropenem synergistically inhibited biofilm formation in CRAB, reducing EPS production, surface motility, QS activity and the expression of key biofilm-related genes, including *ompA*, *bfmR*, *bap*, *csuAB*, *abaI* and *abaR* [64]. These findings indicate that changes in biofilm-associated gene expression may serve as useful markers for assessing the anti-biofilm activity of new treatments. Table 3 was prepared based on a PubMed search using the query “*Acinetobacter baumannii* AND biofilm AND quorum sensing” to summarize the role of QS in the formation, maturation and regulation of *A. baumannii* biofilms.

Overall, Table 2 shows that AbaI/AbaR- and AHL-dependent QS contributes to several stages of *A. baumannii* biofilm development. The selected studies indicate that QS is involved not only in biofilm biomass formation, but also in surface adaptation, motility, matrix organization and structural maturation of the biofilm.

The contribution of QS to *A. baumannii* persistence may also extend beyond direct regulation of biofilm formation. During desiccation, bacterial cells experience oxidative stress and increased expression of antioxidant enzymes, such as catalase and superoxide dismutase, can improve their survival under these conditions [72,73,74]. One reported mechanism involves the insertion sequence ISAba1 upstream of *katG*, a catalase-encoding gene, which may enhance *katG* expression and increase tolerance to hydrogen peroxide [74]. Importantly, the activity of these antioxidant defences has been linked to QS regulation, suggesting that QS may help coordinate both biofilm-associated behaviour and adaptation to oxidative stress in *A. baumannii* [25,75]. He et al. proposed that Resistance-Nodulation-cell Division (RND)-family efflux pumps, especially AdeFGH, may interact functionally with the QS system by facilitating the export of QS signalling molecules, thereby supporting biofilm formation [57].

### 3.2. QS Role in Surface-Associated Motility, Adhesion and Invasion of Epithelial Cells of A. baumannii

Although *A. baumannii* was historically considered non-motile due to the absence of flagella, recent studies have shown that several clinical and environmental isolates exhibit robust surface-associated motility on low-percentage agar plates. This phenotype is now recognized as an important component of early surface adaptation, facilitating the interaction of *A. baumannii* with abiotic and biotic surfaces and potentially promoting subsequent adhesion and biofilm development [76,77].

Experimental evidence suggests that surface-associated motility should be considered not only as a phenotypic feature of selected isolates, but also as a QS-linked characteristic potentially involved in colonization. Tang et al. examined 80 clinical isolates and reported that only five displayed clear surface-associated motility, whereas the remaining isolates were weakly motile or non-motile [27]. Importantly, all motile strains harboured the *abaI* and *abaR* genes and produced AHL signalling molecules, suggesting an association between the AbaI/AbaR QS system and surface translocation. The same study demonstrated considerable strain-dependent variability in adhesion and invasion of epithelial cells, with QS-positive isolates showing stronger invasive capacity. These findings are consistent with previous observations showing that disruption of *abaI* markedly reduced motility, whereas exogenous OHC12-HSL restored motility in the *abaI* mutant [27,78]. Collectively, these data support the role of AbaI/AbaR-mediated QS in regulating surface-associated motility and suggest that this system may contribute to epithelial adhesion and invasion, both of which are critical for *A. baumannii* colonization and nosocomial infection [78,79]. More recent findings by Jiang et al. further support this conclusion at the functional level. Using an *abaI* deletion mutant, the authors showed that disruption of QS reduced the ability of *A. baumannii* to adhere to and invade A549 epithelial cells. Infection with the Δ*abaI* strain was also associated with higher host–cell viability, reduced cellular injury, lower production of inflammatory cytokines and decreased apoptosis compared with the wild-type strain [80]. The contribution of the AbaI/AbaR system to surface-associated motility was further demonstrated by Sun et al. in *A. baumannii* ATCC 17978 [38]. In this study, deletion of *abaI*, *abaR*, or both genes markedly impaired bacterial motility on low-percentage agar, indicating that an intact QS system is required for efficient surface translocation. Interestingly, complementation of the *abaR* mutant partially restored motility, whereas complementation of the *abaI* mutant did not recover the wild-type phenotype. Moreover, overexpression of *abaR* increased motility compared with the wild-type strain. These findings suggest that both components of the AbaI/AbaR system participate in motility regulation, with AbaR potentially exerting a stronger or more direct regulatory effect on this phenotype [38]. However, AbaI/AbaR-mediated regulation should not be interpreted as a linear or isolated process, because this QS system interacts with additional regulatory networks, for example, the Biofilm formation Regulator/Sensor (BfmRS) two-component system [81].

Therefore, the regulatory and phenotypic alterations described above may serve as useful indicators for assessing the efficacy of QQ approaches targeting AbaI/AbaR-mediated signalling. Mayer et al. demonstrated that a functional AbaI/AbaR QS system is required for surface-associated motility in *A. baumannii* ATCC 17978. Both deletion of the AHL synthase gene *abaI* and enzymatic degradation of AHL signals by the QQ enzyme Aii20J reduced surface-associated motility, indicating that AHL-mediated QS contributes to the transition of *A. baumannii* from planktonic growth to surface-adapted phenotypes [34].

### 3.3. Drug Resistance and Stress Adaptation

The contribution of QS to antibiotic resistance has been examined using QS-deficient mutants. Sun et al. [38] showed that the deletion of *abaI*, *abaR*, or both QS genes in *A. baumannii* ATCC 17978 reduced the minimum inhibitory concentration (MIC) values of several antibiotics like kanamycin, gentamicin, penicillin, streptomycin, meropenem, imipenem and ampicillin, compared with the wild-type strain. Complementation of *ΔabaI* and *ΔabaR* partially restored the wild-type susceptibility pattern, whereas overexpression of *abaI* or *abaR* increased the MICs of selected antibiotics due to enhanced biofilm formation. In particular, WT(pMEabaI) with an additional *abaI* copy showed higher MICs for cefepime and cefoperazone–sulbactam, while WT(pMEabaR) with an additional *abaR* copy showed higher MICs for kanamycin, streptomycin, ceftizoxime, cefepime, cefoperazone–sulbactam and piperacillin–tazobactam. These findings suggest that QS-mediated modulation of antimicrobial susceptibility in *A. baumannii* is both gene-specific and antibiotic-dependent [38]. Mendes et al. further linked QS with antibiotic adaptation by showing that a ciprofloxacin-resistant isogenic strain of *A. baumannii* ATCC 19606 exhibited increased biofilm formation and overexpression of the AbaI/AbaR QS system in biofilm cells. Importantly, QS inhibition reduced biofilm formation without changing ciprofloxacin MIC, suggesting that QS may participate in the co-regulation of antibiotic resistance, biofilm development and virulence rather than acting as the sole determinant of resistance [82]. The link between AbaI/AbaR-mediated QS and antimicrobial resistance is also supported by data from clinical *A. baumannii* isolates. Previous findings indicate that the OHC12-HSL AHL signal, produced by *A. baumannii*, can enhance the expression of resistance-associated genes, including *blaOXA-51*, *blaAmpC*, *adeA* and *adeB* [83]. In a collection of 80 clinical strains, most isolates showed high resistance rates to commonly used antibiotics and 75% were classified as MDR. The presence of both *abaI* and *abaR* genes was detected in a substantial proportion of isolates and a subset of these strains produced detectable AHL signalling molecules [27].

However, this effect appears to be strain- and antibiotic-dependent. In contrast to Sun et al. [38], Xiong et al. [41] showed that *abaI* deletion in *A. baumannii* ATCC 19606 strongly affected the virulence-related pathways but did not cause a clear global change in antimicrobial resistance. This study is important because it indicates that the impact of AbaI/AbaR on antimicrobial resistance may be both strain-dependent and antibiotic-specific [41].

Subinhibitory antibiotic concentrations may arise in clinical settings as a consequence of inadequate dosing, limited penetration into infected tissues, biofilm-associated barriers, or pharmacokinetic conditions that prevent effective drug exposure at the infection site. At these levels, antibiotics may fail to eliminate *A. baumannii* and instead function as stress-related signals capable of altering bacterial physiology. In this context, it has been shown that exposure to sub-MICs of meropenem or tigecycline can affect AbaI/AbaR-mediated QS. In particular, increased expression of *abaI* and *abaR* under subinhibitory antibiotic pressure was positively associated with biofilm formation, suggesting that QS may participate in the adaptive response of *A. baumannii* to non-lethal antibiotic exposure [84]. This association was further supported by functional evidence provided by Jiang et al., who directly investigated the role of AbaI during antibiotic-induced adaptation. It has been shown that sub-MIC meropenem pressure can promote an adaptive phenotype in *A. baumannii* through an AbaI-dependent mechanism. Under antibiotic stress, the wild-type strain displayed increased antimicrobial resistance, biofilm formation, surface-associated motility, adhesion and invasion of A549 cells, as well as enhanced virulence in the *Galleria mellonella* model. In contrast, the Δ*abaI* mutant did not show comparable increases in resistance, motility, host–cell interaction, or pathogenicity, indicating that AbaI-mediated QS contributes to antibiotic-induced virulence adaptation [50].

AbaI-mediated QS may also contribute to antimicrobial resistance and stress adaptation through mechanisms extending beyond biofilm regulation. Wang et al. showed that CRISPR-Cas-mediated repression of endogenous *abaI* in the clinical isolate AB43 was associated with reduced efflux pump activity and lower drug resistance, suggesting that AbaI-dependent QS may participate in antimicrobial resistance partly through efflux pump regulation [85]. The same study also linked reduced *abaI* activity with increased reactive oxygen species (ROS) accumulation, indicating that active QS may help *A. baumannii* limit antibiotic-induced oxidative stress [85].

Additional evidence suggests that *abaI* influences membrane-associated adaptation. Pumirat et al. demonstrated that the Δ*abaI* mutant displayed impaired outer membrane structure and increased susceptibility to membrane-targeting antibiotics. Moreover, despite the upregulation of a metallo-β-lactamase-superfamily protein, the *ΔabaI* mutant showed lower MICs for the carbapenems imipenem and meropenem. These findings indicate that AbaI affects antimicrobial susceptibility through multiple and potentially overlapping mechanisms, including outer membrane integrity, envelope adaptation and resistance-associated protein expression [49]. Together, these observations support the view that AbaI/AbaR-mediated QS contributes to antimicrobial resistance and stress adaptation in *A. baumannii* by coordinating efflux activity, oxidative stress control, membrane integrity and biofilm-associated antibiotic tolerance.

## 4. AbaI/AbaR as a Mediator of Host–Pathogen Interactions

During infection, *A. baumannii* adheres to and invades epithelial surfaces, promotes tissue injury and interacts with innate immune defences [86,87,88,89].

The host–pathogen relevance of the AbaI/AbaR QS system can be interpreted at two levels. The better-supported mechanism involves AbaI/AbaR-dependent regulation of bacterial behaviour during infection [27,34,38,49,50,59,85]. A second, less directly demonstrated possibility is signalling between *A. baumannii* and host, in which diffusible AHL molecules influence host–cell pathways [80,87]. However, in *A. baumannii*, current evidence does not clearly distinguish direct effects of AHLs on host cells from indirect consequences of QS-regulated bacterial phenotypes [80].

### 4.1. Host Consequences of AbaI/AbaR-Regulated Virulence Traits

As described in Section 3, AbaI/AbaR-dependent QS has been associated with bacterial phenotypes that are relevant to host interaction, including biofilm formation, surface-associated motility, adhesion and invasion of epithelial cells, antibiotic susceptibility, and stress adaptation [27,34,38,49,50,59,85]. In the context of infection, these traits are important because they influence the ability of *A. baumannii* to attach to host-associated surfaces, persist in protected bacterial communities, interact with epithelial cells, and maintain fitness under hostile conditions such as antimicrobial exposure or host-derived stress [7,59,60,61]. Therefore, the host–pathogen relevance of AbaI/AbaR should be interpreted mainly through its effect on bacterial behaviour that facilitates colonisation and persistence.

Host-model evidence supports this interpretation. Among clinical *A. baumannii* isolates, the presence of a detectable AHL-producing QS phenotype was associated with stronger invasion of A549 epithelial cells and higher virulence in the *Galleria mellonella* infection model [27]. In *A. baumannii* ATCC 17978, disruption of *abaI* markedly reduced virulence in *G. mellonella* and attenuated lethality and early bacterial burdens in a murine infection model, whereas deletion of *abaR* did not produce the same reduction in virulence [38]. Under sub-inhibitory meropenem pressure, the wild-type strain showed increased biofilm formation, surface-associated motility, adhesion and invasion of A549 cells, and enhanced virulence in *G. mellonella* and mice, while the *ΔabaI* mutant did not show comparable antibiotic-induced increases [50]. These more direct findings indicate that AbaI-dependent QS contributes to host colonisation and infection outcome through regulation of virulence-associated bacterial traits.

### 4.2. Epithelial Injury and Apoptosis

Evidence from other bacterial species demonstrates that AHLs can directly affect epithelial cells. Direct exposure to the *P. aeruginosa* signal 3-oxo-C12-homoserine lactone (3O-C12-HSL) disrupted junction-associated proteins and increased paracellular permeability in Caco-2 epithelial monolayers, while inducing mitochondrial dysfunction, reactive oxygen species production, and apoptosis in LS174T epithelial cells [88,89,90]. It should be noted that both cell types are derived from colorectal epithelial cancer cell lines. These findings do not concern *A. baumannii* and should therefore be considered only as proof of principle that an AHL can directly affect host epithelium.

In A549 cells, infection with the ATCC 17978 Δ*abaI* mutant caused less cytoskeletal disruption, LDH release, intracellular ROS, apoptosis, and tumour necrosis factor alpha (TNF-α), interleukin-1 beta (IL-1β), interleukin-6 (IL-6), and interleukin-8 (IL-8) production than the wild-type strain, while host–cell viability was higher. Exogenous AHL supplementation shifted these responses toward the WT phenotype, supporting a contribution of AbaI-dependent signalling to epithelial injury and inflammatory activation [80]. Together, these observations support a functional contribution of AbaI-dependent AHL signalling to epithelial injury, oxidative stress, apoptosis, and inflammatory activation during *A. baumannii* infection [80].

Nevertheless, the experimental design does not distinguish between a direct effect of *A. baumannii*-derived AHLs on epithelial cells and an indirect consequence of AHL-dependent regulation of bacterial phenotypes [80]. Further investigation is required to determine a detailed model of epithelial injury connected to the AbaI/AbaR QS system.

### 4.3. Innate Host Defence During AbaI-Dependent Infection

Evidence connecting AbaI-dependent QS with innate immunity comes mainly from infection models rather than from isolated immune-cell assays. In the murine model used by Sun et al., *ΔabaI* and *ΔabaIR* mutants showed reduced early bacterial burdens in blood, lung, and spleen compared with the wild-type strain, and *ΔabaI* was more susceptible to normal human serum [38]. These findings possibly link AbaI-dependent QS with bacterial survival during early host defence, including serum-dependent antibacterial activity. However, the study did not identify the specific serum component or immune mechanism responsible for this phenotype.

The epithelial cytokine response described in Section 4.2 also has relevance in terms of innate host defence. In the A549 infection model, AbaI-dependent QS was associated with increased production of TNF-α, IL-1β, IL-6, and IL-8, mediators that can amplify local inflammation and promote recruitment or activation of innate immune cells. The zebrafish model used by Jiang et al. additionally reported that the *ΔabaI* mutant showed reduced pathogenicity and reduced neutrophil recruitment compared with the wild-type strain [80]. This result suggests that AbaI-dependent QS might affect the intensity of innate immune recruitment during infection.

### 4.4. Adaptive Immunity: Current Evidence Gap

Current evidence indirectly and/or directly links AbaI-dependent signalling mainly with epithelial responses, serum susceptibility, neutrophil recruitment, and infection outcome, whereas direct effects on dendritic cells (DCs), T cells, B cells, antibody responses, or immunological memory have not been demonstrated for *A. baumannii*. Studies in other Gram-negative pathogens, particularly *P. aeruginosa*, indicate that AHLs can suppress T-cell differentiation and cytokine production, interfere with DC-induced T-cell proliferation, and promote regulatory T-cell induction by preventing DC maturation [91,92,93]. However, these findings should be treated only as proof of principle and cannot be directly extrapolated to the AbaI/AbaR system.

The evidence linking AbaI/AbaR-dependent QS with host-related effects is summarised in Table 4, with emphasis on experimentally observed outcomes and the main limitations of their interpretation.

## 5. QQ and Counter Regulation

### 5.1. Endogenous AbaI/AbaR QQ Enzymes in A. baumannii

Endogenous QQ mechanisms in *A. baumannii* constitute an additional counter-regulatory layer of the AbaI/AbaR QS system [25,31,33]. In contrast to AbaM, which limits the activity of the system at the level of gene expression, QQ mechanisms are based on enzymatic signal quenching, acting directly on AHLs through the degradation or inactivation of signal molecules [25,33,42,55]. Therefore, endogenous QQ enzymes may influence the duration, spatial localization, and accumulation/intensity of the AHL-dependent signal [25,33].

Important evidence for the existence of such mechanisms is provided by Mayer et al. 2018 studies on strain ATCC 17978 [33]. Mayer et al. demonstrated that the main AbaI-dependent signal OHC12-HSL did not accumulate indefinitely. Under static culture conditions, the concentration of the above-mentioned AHL increased, reaching its maximum value during the transition to the stationary phase, and subsequently, markedly decreased. The decrease itself does not directly demonstrate the activity of QQ mechanisms; however, functional assays showed that cell extracts from the examined strain degraded AHLs, which indicated the presence of endogenous activity degrading signal molecules [33]. The above observation can therefore be described as endogenous signal degradation, although the contribution of individual enzymes to this decrease requires additional confirmation [25,33].

The first endogenous QQ enzyme identified in clinical isolates of *A. baumannii* was AidA, classified as an α/β-hydrolase exhibiting AHL-degrading activity. The presence of this enzyme was demonstrated during the analysis of QS-system activity and QQ mechanisms in clinical strains, and the function of AidA was confirmed through heterologous expression in *E. coli* [94]. It should also be remembered that the presence of AidA does not exclude the existence of other QQ enzymes [33,94].

In strain ATCC 17978, both the presence of AidA and additional AHL-degrading activity, not attributable solely to AidA, were demonstrated. Additionally, related *Acinetobacter* strains may exhibit QQ activity even in the absence of an identical *aidA* gene. The above observations support the view that endogenous AHL turnover within the ACB complex depends on several enzymes rather than on a single universal QQ factor [33].

Further analyses of strain ATCC 17978 allowed the identification of several putative AHL lactonases, most of which were classified as proteins belonging to the metallo-β-lactamase-like family. Among the above candidates, the activity of three recombinant enzymes was experimentally confirmed: A1S_2662/YtnP, A1S_0383, and A1S_1876. Together with AidA, these enzymes degraded AHLs in vitro, although their substrate ranges were not identical. Additionally, cell extracts from ATCC 17978 preferentially degraded long-chain AHLs and reduced OHC12-HSL levels by approximately 75% within 3 h. These findings indicate that endogenous QQ activity in *A. baumannii* acts on the predominant AHL form but is not limited to it and may degrade minor AHL fractions [33].

The available literature also presents endogenous QQ mechanisms as condition-dependent processes [33,94]. The expression of *aidA* in strain ATCC 17978 was increased under static culture conditions. In turn, changes in the expression of other putative lactonases were not uniform, and no single defined model emerged from them [33]. Endogenous enzymatic QQ mechanisms, together with AbaM-dependent regulation, may be factors responsible for limiting AHL signal accumulation [33,42,55,94]. This may help explain why the presence of *abaI* and *abaR* does not always mean the display of a single pattern of AHL accumulation or the development of an AHL-dependent phenotype [27,33,42,94]. Irrespective of the above, data on QQ in *A. baumannii* remain incomplete, and most experimental data come from strain ATCC 17978 and selected clinical isolates [25,33,94].

### 5.2. Exogenous AHL-Degrading QQ Enzymes

Numerous strategies have been developed to disrupt QS systems, particularly those targeting biofilm formation. In *A. baumannii*, non-enzymatic QS inhibitors have been investigated mainly as small molecules that interfere with AHL-mediated signalling rather than directly degrading the signal. The most direct evidence concerns non-native AHL analogues acting as AbaR antagonists, which attenuate AbaR-dependent responses and reduce QS-associated phenotypes, including surface-associated motility and biofilm formation [95]. Other compounds, such as virstatin, unsaturated fatty acids, and subinhibitory concentrations of streptomycin, have also been reported to inhibit biofilm formation, motility, or QS-associated signalling in *A. baumannii*, although their mechanistic connection with the AbaI/AbaR system appears less direct or more context-dependent [96,97,98]. Among anti-QS approaches, the enzymatic degradation of AHLs by acylases and lactonases has received particular attention as a promising alternative to these non-enzymatic inhibitors [99,100,101].

The broad-spectrum N-acylhomoserine lactonase Aii20J, derived from the marine bacterium *Tenacibaculum* sp. 20J, has been evaluated as an exogenous QQ agent against *A. baumannii* [34]. By degrading N-acylhomoserine lactone signals involved in AbaI/AbaR-mediated QS, Aii20J blocked surface-associated motility of strain ATCC 17978, while deletion of the N-acylhomoserine lactone synthase gene *abaI* similarly impaired this phenotype. Aii20J also reduced biofilm biomass by approximately 80% in ATCC 17978. However, its activity against MDR clinical isolates was strain-dependent, with significant biofilm inhibition observed in only two of the four isolates tested. The lower sensitivity of isolates producing negligible amounts of N-acylhomoserine lactones suggests that enzyme efficacy may partly depend on endogenous signal production. Transmission electron microscopy showed that Aii20J-treated cells were almost completely devoid of the short surface appendages present in untreated cells, a finding consistent with, but not directly demonstrating, reduced production of chaperone/usher pili. Combined treatment with Aii20J and deoxyribonuclease (DNase) reduced biofilm formation in all five strains examined, although a statistically significant synergistic interaction was not demonstrated. These results support the potential of Aii20J, particularly in combination with matrix-degrading enzymes, for controlling QS-associated motility and biofilm formation in *A. baumannii* [34].

Likewise, an engineered QQ lactonase variant carrying the E101G/R230C substitutions, derived from *Geobacillus kaustophilus*, has also demonstrated strong antibiofilm activity against the clinical *A. baumannii* isolate S1. This enzyme hydrolyses the primary AHL signalling molecules of the bacterium’s LuxI/LuxR-type QS network, namely OHC10-HSL and OHC12-HSL. Quantitative analyses indicate that treatment with the engineered lactonase significantly decreases biofilm biomass, average thickness, and surface coverage. By destabilizing the protective biofilm architecture, this antivirulence approach not only reduces pathogen persistence but may also potentiate the efficacy of conventional antibiotics against *A. baumannii* [102].

Another lactonase, the marine-derived N-acylhomoserine lactonase MomL, originally identified in *Muricauda olearia*, reduced the biofilm biomass of *A. baumannii* LMG 10531 by approximately 42% at 5 μg/mL and enhanced the activity of several antibiotics against biofilm-associated cells. Its combination with tobramycin reduced viable cell numbers by approximately 80% compared with tobramycin alone. However, its activity was strain and model-dependent: a higher concentration was required for LMG 10520, no significant effect was observed for AB5075, and MomL was ineffective in a wound-like biofilm model and a *Caenorhabditis elegans* infection model [103].

AaL, a broad-spectrum N-acylhomoserine lactonase from *Alicyclobacillus acidoterrestris*, belongs to the metallo-β-lactamase superfamily and hydrolyses structurally diverse N-acylhomoserine lactones. In *A. baumannii* ATCC 19606, AaL reduced biofilm formation by more than fourfold relative to untreated cultures without decreasing bacterial cell density, supporting an antibiofilm rather than growth-inhibitory effect. However, its activity has not been evaluated in clinical isolates or infection models [104].

Finally, GcL is a thermostable, broad-spectrum N-acylhomoserine lactonase derived from *Geobacillus caldoxylosilyticus*, currently classified as *Parageobacillus caldoxylosilyticus*. Exogenous GcL inhibited biofilm formation by *A. baumannii* ATCC 19606 in a dose-dependent manner without reducing planktonic cell density. However, the study did not identify the endogenous AHL responsible for this effect or assess the enzyme in clinical isolates or infection models. Therefore, its broader relevance to AbaI/AbaR-dependent phenotypes remains to be established [105].

An additional consideration for the therapeutic development of enzymatic QQ is the possibility of bacterial adaptation to sustained signal degradation. Although direct experimental evidence demonstrating the evolution of resistance to exogenous AHL lactonases in *A. baumannii* is currently limited, several potential mechanisms warrant investigation. These may include changes in the spectrum or relative abundance of produced AHLs, altered regulation of *abaI/abaR* expression, increased endogenous degradation of AHLs, or compensatory changes within the QS regulatory network. The AbaM negative regulator provides one example of how modulation of the QS circuit can substantially alter AHL production and signalling output [42,55]. In addition, the structural diversity of AHLs and the strain-dependent AHL profiles observed in *A. baumannii* may influence the susceptibility of individual QS systems to a given QQ enzyme [33,34]. Consequently, future studies should determine whether prolonged exposure to QQ enzymes selects for altered AHL profiles, changes in QS regulatory activity, or fitness-compensating phenotypes. Such studies will be important for assessing the durability of enzymatic QQ and for designing combination strategies that reduce the likelihood of adaptive escape.

Collectively, these studies demonstrate that enzymatic QQ, particularly through AHL lactonases, represents a promising antivirulence strategy for attenuating QS-dependent phenotypes in *A. baumannii*, including biofilm formation and surface-associated motility. However, the strain-dependent efficacy of these enzymes and the limited validation in clinically relevant infection models highlight the need for further studies to establish their therapeutic potential.

### 5.3. Host-Mediated QS Modulation

Host-derived QQ has not yet been directly demonstrated for *A. baumannii*. Nevertheless, studies involving other Gram-negative pathogens show that mammalian enzymes can degrade bacterial AHLs, providing a plausible basis for investigating host-mediated interference with the AbaI/AbaR system. Human paraoxonases PON1, PON2, and PON3 possess lactonase activity. PON1 and PON3 are produced predominantly in the liver and circulate in association with high-density lipoprotein (HDL), whereas PON2 is a cell-associated enzyme expressed in numerous tissues [106,107]. All three paraoxonases hydrolyse the *P. aeruginosa* signal OC12-HSL, with PON2 exhibiting the highest activity [108]. Accordingly, PON2 deficiency in murine tracheal epithelia impaired OC12-HSL degradation and enhanced *P. aeruginosa* QS, while transgenic expression of human PON1 protected *Drosophila melanogaster* against *P. aeruginosa* in a lactonase-dependent manner [109,110].

However, these findings cannot be directly extrapolated to *A. baumannii*. The available studies concern predominantly *P. aeruginosa* and OC12-HSL, whereas the major AbaI-dependent signal of *A. baumannii* is OHC12-HSL [33]. To date, it has not been demonstrated that host paraoxonases degrade this signal during *A. baumannii* infection or suppress AbaI/AbaR-dependent phenotypes. Similarly, aryl hydrocarbon receptor (AhR)-mediated recognition of *P. aeruginosa* QS metabolites represents host surveillance rather than direct signal quenching and has not been investigated in *A. baumannii* [111]. Additional, less well-defined forms of host-dependent QS modulation may involve host-derived signal mimics and interactions with the resident microbiota. Mammalian epithelial cells can produce an autoinducer-2 (AI-2)-mimicking activity in response to bacterial exposure or disruption of epithelial tight junctions, while commensal microorganisms may influence QS through signal production, cross-species signalling, or signal degradation [112,113]. However, these observations concern signalling networks other than the AbaI/AbaR system, and their relevance to AHL-dependent communication in *A. baumannii* remains unknown [87,112]. Host-mediated degradation or sensing of *A. baumannii* AHLs should therefore be regarded as a plausible but currently unverified research direction requiring direct biochemical studies and validation in infection models.

## 6. Future Directions

Despite the growing interest in anti-virulence therapies and the increasing recognition of QS as an important regulator of *A. baumannii* pathogenicity, the clinical translation of QS-targeted strategies remains in its infancy. This is largely due to the scarcity of large-scale studies and lack of relevant preclinical validation. In *A. baumannii*, the AbaI/AbaR regulatory network, including the modulator AbaM, has emerged as an important determinant of pathogenesis-related traits and antimicrobial adaptation. However, current mechanistic understanding still relies heavily on AbaI mutant analyses, in vitro phenotypic assays, and a limited number of laboratory or strain-specific models, whereas direct definition of the AbaR regulon and its downstream effectors remains incomplete. This limitation has important translational implications, as QS-associated phenotypes vary substantially across clinical isolates, and the presence of AbaI or AbaR alone does not reliably predict AHL production, biofilm formation, antimicrobial tolerance, or virulence potential.

The translational potential of QQ may also be enhanced by combining signal interference with other anti-infective approaches. The available evidence already provides examples of potentially complementary strategies: Aii20J reduced biofilm formation and surface-associated motility, while its combination with DNase reduced biofilm formation across the strains examined, although a statistically significant synergistic interaction was not demonstrated [34]. Similarly, MomL enhanced the activity of several antibiotics against biofilm-associated cells, with its combination with tobramycin reducing viable cell numbers compared with tobramycin alone [103]. These observations support further investigation of QQ enzymes in combination with conventional antibiotics and matrix-targeting approaches. Other combinations, including QQ with bacteriophage therapy, antimicrobial peptides, or nanoparticle-based delivery systems, represent additional research directions because simultaneous disruption of QS-regulated behaviours and bacterial viability or biofilm structure could potentially improve treatment efficacy. However, such combinations should currently be regarded as prospective strategies rather than established therapeutic regimens, and their efficacy, compatibility, pharmacokinetics and potential for adaptive bacterial responses require experimental validation.

Future research should therefore prioritize comprehensive mapping of the AbaR regulon by integrating transcriptomic, proteomic and single-cell approaches with clinically diverse isolate collections to define conserved QS-dependent pathways and identify robust therapeutic targets. Equally important will be the evaluation of QQ enzymes, small-molecule QS inhibitors, and other anti-virulence agents in combination with conventional antibiotics or immunomodulatory therapies. Such combination strategies may enhance bacterial clearance while reducing tissue damage and limiting the selective pressure associated with traditional antimicrobial therapy. Ultimately, successful clinical translation will require standardized susceptibility testing for QS inhibitors, validated biomarkers of QS activity in vivo, and well-designed preclinical and clinical studies demonstrating efficacy in clinically relevant models of CRAB infection.

## 7. Methods

A comprehensive literature search was conducted using PubMed, Google Scholar, and Scopus databases. Advanced search strategies primarily targeted studies published between 2021 and 2026 to capture recent developments in the field, although earlier pivotal publications were included when deemed relevant. Search queries were constructed using combinations of Medical Subject Headings (MeSH) and free-text terms/keywords, including “*Acinetobacter baumannii*”, “quorum sensing”, “biofilms”, “drug resistance, bacterial”, “virulence”, “host-pathogen interactions”, “innate immunity”, “AbaI”, “AbaR”, “AbaI/AbaR”, “N-acyl homoserine lactone”, “stress adaptation”, “quorum quenching”, “quorum sensing inhibitors”, and “anti-virulence strategies”. While advanced search methods prioritized the literature from the past five years, this review analyses research spanning from 1999 to 2026. Inclusion criteria were: (1) peer-reviewed original research articles about QS and QQ; (2) systematic and narrative reviews published in peer-reviewed journals and relevant to QS and QQ in *A. baumannii* and (3) articles published in English. Exclusion criteria were: (1) non-peer-reviewed reports and other publications lacking formal peer review; (2) conference abstracts without full-text availability; and (3) publications of insufficient scientific quality or relevance to the scope of this review. The tables and figures presented in this review were created using Autodesk Sketchbook, version 6.2 (released 23 May 2025), and GoodNotes 5 (v5.9.117; Goodnotes Limited, London, UK) on the iPadOS platform. They were prepared through synthesis of information from the selected literature to provide an integrated overview of the key concepts and findings discussed.

## 8. Conclusions

The *abal/abaR* genotype alone cannot reliably indicate active QS. Gene presence does not guarantee detectable AHL production, nor does AHL detection ensure consistent phenotypic outcomes across *A. baumannii* isolates. Distinguishing genetic potential from functional QS activity requires integrated approaches combining molecular detection, direct AHL measurement, and phenotypic characterization. Developing standardized QS-activity biomarkers is essential for advancing AbaI/AbaR-targeted therapeutic strategies.

The AbaI/AbaR system’s role in pathogenesis appears multifactorial, operating through both direct and indirect mechanisms affecting bacterial virulence and host interactions. However, the system’s contribution to infection cannot be fully understood without accounting for additional regulatory factors and strain-specific variations. Future research should prioritize distinguishing direct QS-mediated effects from indirect consequences of genetic background, particularly when evaluating therapeutic interventions targeting this pathway.

## Figures and Tables

**Table 1:** AHL Signals Discussed in the AbaI/AbaR QS System of *A. baumannii.*

Abbreviation	Chemical Name	Structural Characteristic
C6-HSL	N-Hexanoyl-L-homoserine lactone	C6, unsubstituted
OC6-HSL	N-(3-Oxohexanoyl)-L-homoserine lactone	C6, 3-oxo
C8-HSL	N-Octanoyl-L-homoserine lactone	C8, unsubstituted
OHC10-HSL	N-(3-Hydroxydecanoyl)-L-homoserine lactone	C10, 3-hydroxy
OHC12-HSL	N-(3-Hydroxydodecanoyl)-L-homoserine lactone	C12, 3-hydroxy
OC12-HSL	N-(3-Oxododecanoyl)-L-homoserine lactone	C12, 3-oxo
OHC14-HSL	N-(3-Hydroxytetradecanoyl)-L-homoserine lactone	C14, 3-hydroxy
OC14-HSL	N-(3-Oxotetradecanoyl)-L-homoserine lactone	C14, 3-oxo

**Table 2:** Summary of key experimental backgrounds used to define the AbaI/AbaR QS system in *Acinetobacter baumannii* and related *Acinetobacter* strains.

Experimental Model	Taxonomic Relevance	Main Contribution to AbaI/AbaR System Knowledge	Main Limitation	References
M2	Historical, currently classified as *A. nosocomialis*, part of closely related *A. calcoaceticus*-*A. baumannii* complex	Foundational identification of AbaI-dependent AHL production; OHC12-HSL-centered profile; AHL-dependant AbaI induction	Cannot be treated as species-specific evidence for *A. baumannii*	[32,33,40]
ATCC 17978 WT and *ΔabaI* mutant	Confirmed *A. baumannii* reference strain	OHC12-HSL-centered profile; AbaI disruption abolishes detectable AHL production; AHL accumulation depends on culture conditions; endogenous QQ activity described	Laboratory reference strain, may not represent clinical lineages	[33,34,49]
ATCC 19606 WT and *ΔabaI* mutant	Confirmed *A. baumannii* reference strain	Provided evidence that *ΔabaI* affects broad transcriptional programs, including metabolism, biofilm-associated genes and T6SS-related genes	Differences in expression don’t prove direct AbaR influence on all affected genes	[41]
AB5075 WT and *abaI* and *abaM* mutants	Confirmed *A. baumannii* reference strain; clinically relevant	Provided evidence for AbaM as RsaM-like negative modulator; *abaM* disruption increases AHL production and affects ~21% of QS system regulon	Differences in expression don’t prove direct AbaM influence on all affected genes	[42]
Clinical isolates	Clinically relevant *A. baumannii* isolate collection	Demonstration of strain variability; presence of *abaI* and *abaR* does not always predict detectable AHL production; detectable AHL production does not correspond to a uniform phenotype	Heterogeneous backgrounds and aggregate analyses may limit direct strain-to-strain comparison	[27,33]

Abbreviations: *A. baumannii—Acinetobacter baumannii*; *A. nosocomialis—Acinetobacter nasocomialis*; *A. calcoacetius—Acinetobacter calcoacetius*; AHL—acyl-homoserine lactone; WT—wild-type; ATCC—American Type Culture Collection; OHC12-HSL—N-(3-hydroxydodecanoyl)-L-homoserine lactone; QQ—quorum quenching; QS—quorum sensing; T6SS—type VI secretion system.

**Table 3:** Experimental evidence for the involvement of quorum sensing (QS) in *Acinetobacter baumannii* biofilm formation and maturation.

QS Component	*A. baumannii* Strain	Key Findings	References
AHL-dependent QS	*A. baumannii* ATCC 17978 WT strain, *abaI* deletion mutant and *csuD*::*kan* insertion mutant	The *abaI* mutant formed only scattered monolayers, whereas the WT strain developed thick mushroom-shaped microcolonies; supplementation with AHLs, especially OHC10-HSL, restored complex biofilm architecture.	[68]
AbaI-dependent QS	*A. baumannii* ATCC 19606 WT strain and Δ*abaI* mutant	Deletion of *abaI* reduced biofilm formation in *A. baumannii* ATCC 19606, indicating that AbaI-dependent QS contributes to biofilm-associated pathogenicity.	[41]
AbaI/AbaR QS	*A. baumannii* ATCC 17978; Δ*abaI*, Δ*abaR* and Δ*abaIR* mutants	In comparison with the WT strain, biofilm production was markedly reduced in most mutant and complemented strains, whereas *abaR* overexpression in WT(pMEabaR) enhanced biofilm formation beyond the WT level.	[38]
AbaI-mediated QS	*A. baumannii* ATCC 17978; Δ*abaI and ΔabaI::abaI*	Deletion of *abaI* significantly reduced biofilm biomass compared with the WT strain, while genetic complementation of *abaI* restored biofilm formation to a wild-type-like level.	[49]
CRISPR-Cas–AbaI axis	Clinical isolate AB43 with a complete I-Fb CRISPR-Cas system and deletion mutants	Loss of the CRISPR-Cas system in the clinical *A. baumannii* AB43 strain increased biofilm formation, as deletion of the whole *crispr-cas* locus or individual components such as *cas1, cas3* and *csy1–csy4* resulted in stronger biofilm production than the WT or complemented strains.	[69]
AbaI/R and biofilm-associated gene expression	*A. baumannii* strains, KBN10P01317 (low-virulent) and KBN10P01599 (high-virulent)	Although the two *A. baumannii* strains carried a comparable set of virulence-associated genes, strain KBN10P01599 showed markedly higher expression of *abaI/abaR* and key biofilm-related genes, including *csuCDE*, *bap* and *pgaA*, which was associated with enhanced surface motility, stronger biofilm formation and increased adhesion to host cells.	[70]
DksA–AbaI/AbaR regulatory interaction	*A. baumannii* ATCC 17978 WT, Δ*dksA* mutant and complemented strain	DksA modulates QS and biofilm regulation in a non-linear manner: Δ*dksA* reduced autoinducer production and *abaI/abaR* expression, but increased biofilm formation and upregulated *BfmR/S* and *csuC/D/E*-related genes.	[71]

Abbreviations: *A. baumannii*—*Acinetobacter baumannii*; QS—quorum sensing; AHL—acyl-homoserine lactone; WT—wild-type; ATCC—American Type Culture Collection; OHC10-HSL—N-(3-hydroxydecanoyl)-L-homoserine lactone; CRISPR-Cas—clustered regularly interspaced short palindromic repeats and CRISPR-associated proteins; MIC—minimum inhibitory concentration; MBEC—minimum biofilm eradication concentration; XDR—extensively drug-resistant.

**Table 4:** Level of evidence for AbaI/AbaR-dependent host-related effects.

Host-Related Effect	Experimentally Observed Outcome	Experimental Model	Main Limitation	Key References
Adhesion/invasion of epithelial cells	*ΔabaI* or AHL-producing phenotype changes adhesion/invasion in A549 cells	A549 epithelial infection model	Does not distinguish direct AHL-host signalling from general observations	[27,50,80]
Epithelial injury/apoptosis	*ΔabaI* causes less LDH release, ROS, apoptosis and cytokine production; AHL supplementation shifts phenotype toward wild-type	A549 epithelial infection model	Reduced injury may reflect reduced bacterial adhesion/invasion	[80]
Infection outcome	*ΔabaI* shows reduced virulence in *Galleria mellonella*, zebrafish and/or mouse models	*Galleria mellonella*, zebrafish, murine infection models	Model-dependent; *ΔabaR* effects are not equivalent to *ΔabaI*	[27,38,50,80]
Serum susceptibility/early clearance	*ΔabaI* and *ΔabaIR* show reduced early burdens and increased serum sensitivity	Normal human serum assay; murine early-burden model	Specific serum component or immune mechanism not identified	[38]
Neutrophil recruitment	*ΔabaI* reduces neutrophil recruitment in zebrafish	Zebrafish infection model	Could reflect lower bacterial burden/virulence rather than direct immune modulation	[80]
Adaptive immunity	No direct AbaI/AbaR *A. baumannii* data	No direct *A. baumannii* AbaI/AbaR data; proof-of-principle AHL studies in *P. aeruginosa*	Only proof-of-principle data from other AHL-producing bacteria	[91,92,93]

Abbreviations: *A. baumannii*—*Acinetobacter baumannii*; *P. aeruginosa*—*Pseudomonas aeruginosa*; AHL—acyl-homoserine lactone; LDH—lactate dehydrogenase; ROS—reactive oxygen species.

## Data Availability

No new data were created or analysed in this study.

## Abbreviations

The following abbreviations are used in this manuscript:

*A. baumannii*	*Acinetobacter baumannii*
*A. calcoaceticus*	*Acinetobacter calcoaceticus*
*A. nasocomialis*	*Acinetobacter nasocomialis*
*P. aeruginosa*	*Pseudomonas aeruginosa*
AHL	acyl-homoserine lactone
AIs	autoinducers
AI-2	autoinducer-2
AbaI	*A. baumannii* LuxI-type autoinducer synthase
AbaR	*A. baumannii* LuxR-type transcriptional regulator
AbaM	RsaM-type negative regulator/modulator of the AbaI/AbaR quorum-sensing system
QS	quorum sensing
QQ	quorum quenching
QSIs	quorum-sensing inhibitors
AHLs	acyl-homoserine lactones
HSL	homoserine lactone
OHC10-HSL	N-(3-hydroxydecanoyl)-L-homoserine lactone
OHC12-HSL	N-(3-hydroxydodecanoyl)-L-homoserine lactone
OHC14-HSL	N-(3-hydroxytetradecanoyl)-L-homoserine lactone
OC12-HSL	N-(3-oxododecanoyl)-L-homoserine lactone
OC14-HSL	N-(3-oxotetradecanoyl)-L-homoserine lactone
OC6-HSL	N-(3-oxohexanoyl)-L-homoserine lactone
C6-HSL	N-hexanoyl-L-homoserine lactone
C8-HSL	N-octanoyl-L-homoserine lactone
WT	wild-type
ATCC	American Type Culture Collection
ACB	*Acinetobacter calcoaceticus–Acinetobacter baumannii* complex
CRAB	carbapenem-resistant *Acinetobacter baumannii*
MDR	multidrug-resistant/multidrug resistance
XDR	extensively drug-resistant
MIC	minimum inhibitory concentration
MBEC	minimum biofilm eradication concentration
HAIs	healthcare-associated infections
HAP	hospital-acquired pneumonia
VABP	ventilator-associated bacterial pneumonia
BSI	bloodstream infection
UTI	urinary-tract infection
ICU	intensive care unit
WHO	World Health Organization
CDC	Centers for Disease Control and Prevention
ROS	reactive oxygen species
LDH	lactate dehydrogenase
EPS	extracellular polymeric substance
T6SS	type VI secretion system
RND	resistance-nodulation-cell division
CRISPR-Cas	clustered regularly interspaced short palindromic repeats and CRISPR-associated proteins
IL-1β	interleukin-1 beta
IL-6	interleukin-6
IL-8	interleukin-8
TNF-α	tumor necrosis factor alpha
DCs	dendritic cells
AhR	aryl hydrocarbon receptor
AbOmpA	*Acinetobacter* outer membrane protein A
OmpA	outer membrane protein A
OmpW	outer membrane protein W
BamA	β-barrel assembly machinery protein A
SurA	survival protein A
Bap	biofilm-associated protein
BfmRS	Biofilm formation Regulator/Sensor two-component system
AdeFGH	AdeFGH efflux pump
PON1	paraoxonase 1
PON2	paraoxonase 2
PON3	paraoxonase 3
ACP	acyl carrier protein
3O-C12-HSL	3-oxo-C12-homoserine lactone
